# Latent Strongyloides stercoralis in an Asymptomatic Male With Chronic Peripheral Eosinophilia

**DOI:** 10.7759/cureus.20140

**Published:** 2021-12-03

**Authors:** Taha F Rasul, Daniel R Bergholz, Arfa Faiz

**Affiliations:** 1 Medicine, University of Miami Miller School of Medicine, Miami, USA; 2 Allergy and Immunology, Sutter Medical Center, Sacramento, USA

**Keywords:** holistic evaluation, guided testing, latent parasitic infection, peripheral eosinophilia, strongyloides sterocalis

## Abstract

Peripheral eosinophilia is a potentially concerning finding that can occur due to a multitude of causes. One such cause is latent helminth infections such as Strongyloides stercoralis. These parasites have broad distributions throughout the developing world, particularly South and Southeast Asia and it is estimated that roughly 200 million people have latent infections. We present the case of a 74-year-old patient from India who had asymptomatic eosinophilia since before 2006. He previously underwent an extensive workup which included testing for neoplasms, gene mutations, and lymphoproliferative disorders. After carefully examining the patient’s travel history and demographic information, a parasite panel was administered which was positive for Strongyloides, thereby establishing a cause for his condition after years of expensive testing. Latent Strongyloides infections can lead to fatal dissemination if the host becomes immunocompromised. It is therefore essential to keep a detailed history of patient travel, occupation, and functional status when assessing peripheral eosinophilia so that obvious causes are not overlooked.

## Introduction

Strongyloidiasis is a condition which results from infection by the helminth Strongyloides stercoralis, which is endemic to rural tropical regions and typically infects patients through skin contact with contaminated soil. This helminth is rather unique due to its ability to complete its life cycle entirely within the host. Adult worms may burrow into the mucosa of the duodenum and jejunum and live there for up to five years. This trait allows it to evade detection for an extended period of time or until the host is immunosuppressed. In healthy individuals, Strongyloides typically cause no overt pathology. The estimated global prevalence is between 70-200 million cases, the majority being in Southeast Asia and Central-West Africa.

Peripheral eosinophilia is defined as a concentration of more than 400-500 eosinophils per microliter of blood and presents a challenge to clinicians due to its wide range of etiologies such as inflammatory, allergic, parasitic, and neoplastic disorders [1-3]. The eosinophil count is not an accurate reflection of its cause. That is, substantial yet equivalent levels of eosinophilia can be seen in neoplastic processes as well as certain drug-hypersensitivity reactions [4].

Therefore, it is imperative to identify the cause of eosinophilia by patient history, presentation, and guided laboratory testing. The occupational, travel, and cultural background of patients can provide a framework to steer management and evaluation. Whereas constitutional symptoms such as fever, unintended weight loss, and night sweats may be indicative of an underlying neoplastic process, other disorders like eczema can present with only cutaneous lesions. Peripheral eosinophilia is often asymptomatic, and often manifests without overt symptoms or organ involvement; it may not become apparent until found with incidental blood-work [5]. The wide etiology of eosinophilia means that patients may be subjected to “shotgun” testing (that is, using the approach of trying several possible solutions simultaneously) or not be evaluated at all.

Asymptomatic eosinophilia requires particular evaluation of travel history [6]. This is because certain parasites such as Strongyloides are found worldwide, including the American Appalachian region. Patients with eosinophilia who travel or immigrate from endemic areas should have a serologic test as a screening measure, before any further workup. Moreover, with expanding immigration and refugee resettlement, these populations can have very subtle symptoms or be completely asymptomatic in the event of parasitic infections, and only have mild eosinophilia. Furthermore, some helminth infections may continue almost indefinitely after acquisition [7]. It is important to note that these parasitic infections often affect immunologically competent individuals and stay latent until they are eventually cleared by the immune system, treated medically, or spread diffusely in the case of immunocompromise. For this reason, uncomplicated cases of eosinophilia in otherwise healthy individuals should be approached by a thorough evaluation of patient background, travel, and other determining factors.

One other important consideration is that patients with latent infections can have a potentially overwhelming resurgence of the parasite in cases where they may be immunocompromised, such as in high-dose corticosteroid therapy [8]. In these cases, the dissemination of parasitic infections in the context of immunomodulatory drugs has been linked to high rates of mortality [9]. Hence, in patients who may have suspected latent parasitic infections, care should be taken prior to initiation of high-dose corticosteroids [10].

For this case, we present a 74-year-old male from India who had been living in the United States for roughly two decades. He was noted to have asymptomatic eosinophilia since at least 2006, initially discovered through routine bloodwork. He was previously tested extensively for hematological, lymphoproliferative, and neoplastic disorders due to his age, and after negative findings throughout, had learned to live with a diagnosis of idiopathic eosinophilia. Other notable comorbidities include hypertension and stage III chronic kidney disease. There was no history of asthma, allergic rhinitis, or chronic obstructive pulmonary disease (COPD) that may have been suggestive of another etiology of the eosinophilia.

By careful evaluation of the patient’s history, occupation, travel patterns, and presentation, it was possible to narrow the cause of his eosinophilia down to a latent, untreated parasitic infection, ostensibly from his country of origin.

## Case presentation

A 74-year-old male of Indian descent was being evaluated for an episode concerning for laryngeal edema which resulted in an emergency room (ER) visit. He had reported that he had dinner in the evening, with the same kind of food that he had been consuming for years. Roughly half an hour later, he started experiencing symptoms of throat swelling and tightening. He then went to bed and was awoken late at night by extreme discomfort in his throat which made him feel like he could not breathe. Taking cetirizine alleviated the symptoms briefly, but he was promptly taken to the ER. Physical examination showed no swelling, and treatment became centered around pharyngitis for which he received dexamethasone. However, he still had the feeling in his throat for the next two days. His scheduled lisinopril had been discontinued to prevent further exacerbation of the symptoms due to the increased risk of angioedema from angiotensin-converting enzyme (ACE) inhibitors.

After this episode, he recovered slowly, and by the time he presented to the allergy and immunology clinic, he still had residual symptoms and subjectively felt unwell. He was particularly interested in better understanding any potential underlying conditions that may have caused the throat discomfort and his lingering condition. Most notably, he was also being evaluated for peripheral eosinophilia, which had been occurring since 2006. As of January 2021, his eosinophil count was approximately 2400 cells/µL, well above the standard range of 0-500 cells/µL. He was being treated for hypertension by his primary care provider, and his medical history included multiple tests based on his peripheral eosinophilia. There was a concern of some neoplastic process, especially due to his advanced age. An extensive work-up was done whereby he was tested for gastrointestinal disorders, vasculitides, autoimmunity, and lymphoproliferative disorders. Further evaluation for hematological disorders like chronic myeloid leukemia (CML), platelet-derived growth factor receptor alpha (PDGFRA), platelet-derived growth factor receptor beta (PDGFRB), fibroblast growth factor receptor 1 (FGFR1), and systemic mastocytosis were also negative. Finally, antinuclear antibody (ANA) and peripheral blood flow cytometry were found to be noncontributory.

After a benign review of systems, physical examination was notable for retropharyngeal cobblestoning and post-nasal drip. A closer evaluation of the patient’s demographic profile and his immigration status from India meant that there was a possibility of a clinically latent parasitic infection. Sebastian et al. found that among asymptomatic individuals in various parts of southern and northern India, the prevalence of Strongyloides ranged from 20% to higher than 90% [9]. The decision was then made to test for parasitic infections, and Strongyloides O&P (ova and parasite), as well as anti-Strongyloides IgG, were both positive. After prompt treatment with Ivermectin, repeat serology at six months showed eosinophil counts within normal limits.

## Discussion

The fundamental lessons from this case are two-fold. Firstly, it is important to keep parasitic infections high on the differential for individuals from areas where Strongyloides, Ascaris lumbricoides, and Trichuris trichiura are endemic. Our patient had an extensive workup for potential hematological, neoplastic, gastrointestinal, and autoimmune causes of his eosinophilia. This testing, although seemingly necessary at the time, could have been avoided by evaluating the most likely risk factors based on his demographic information. Sebastian et al. also found that the lifetime prevalence of parasitic infections in southern India could be as high as 97% [9]. Therefore, the patient could have avoided excessive testing and a reduced risk of overtreatment [11]. It is also important to note that the psychological impact of overtesting can be very deleterious to the patient and inevitably leads to misallocation of funds and resources [12]. Hence, it is highly recommended in patients with significant histories of living abroad in parasite-endemic areas to be tested for parasitic infections before any further workup [13]. In a “swiss-cheese” model of escalating testing for undetermined peripheral eosinophilia, the first few layers should include parasite testing, keeping patient history in mind.

Latent parasitic infections can cause a superinfection if the patient is in any way immunocompromised. Thomas and Costello mention how latent Strongyloides became disseminated from a single dose of dexamethasone before stereotactic radiosurgery [13]. In that case, the sudden and overwhelming strongyloidiasis was fatal to the patient, and highlighted the need to screen patients with persistent eosinophilia for Strongyloides, particularly before any initiation of high-dose corticosteroids [13]. Our patient was treated for his throat swelling in the ER with dexamethasone, and this could have led to a life-threatening resurgence of Strongyloides. In fact, numerous cases have been reported where patients with underlying Strongyloides infections presented with asymptomatic eosinophilia or respiratory symptoms [14]. Thus, peripheral eosinophilia should be a warning sign before the initiation of any corticosteroids, and adequate care must be taken to rule out any latent parasitic infections that may disseminate upon administration.

## Conclusions

Peripheral eosinophilia, even if asymptomatic, should be followed regularly due to the wide spectrum of potential etiologies, especially in the presence of risk factors like travel history. Patients with asymptomatic eosinophilia from areas where parasitic infection is endemic should be closely evaluated before the initiation of any corticosteroids to prevent a life-threatening parasitic resurgence. Additionally, Strongyloides and other parasitic infections should be kept high on the differential in patients from endemic areas who may otherwise be asymptomatic, in order to avoid any unnecessary further testing.
